# Fatigue Behavior of Inorganic-Organic Hybrid “Lunar Cement”

**DOI:** 10.1038/s41598-019-38799-x

**Published:** 2019-02-19

**Authors:** Hang Su, Youshi Hong, Tzehan Chen, Rui Kou, Meng Wang, Ying Zhong, Yu Qiao

**Affiliations:** 1 State Key Laboratory of Nonlinear Mechanics, Institute of Mechanics, Chinese Academy of Sciences, Beijing, 100190 China; 2 School of Engineering Science, University of Chinese Academy of Sciences, Beijing, 100049 China; 3 Program of Materials Science and Engineering, University of California – San Diego, La Jolla, CA 92093 USA; 4 Department of Structural Engineering, University of California – San Diego, La Jolla, CA 92093-0085 USA

## Abstract

We report the experimental results of fatigue behavior of ultralow-binder-content inorganic-organic hybrid (IOH) based on lunar soil simulant, which may be viewed as a “lunar cement”. Under the same loading condition, the fatigue life of the IOH is superior to typical steel-reinforced concrete, especially when the stress amplitude is relatively high. Fatigue damage mostly occurs in the binder phase, followed by rapid cleavage-like failure. The important material parameters include filler type, filler particle size, and filler-binder bonding.

## Introduction

Crewed lunar exploration missions have been actively studied for decades^1^, for which construction of lunar bases and/or outposts are of immense importance^2^. The first-generation lunar structures are likely based on inflatable and/or self-deploying habitats produced on the Earth^3^. They will be multi-functional. However, due to the tight limitation of space transportation, pre-fabricated lunar habitats have to be small-sized, imposing tough challenges to their multifunctionality and long-term sustainability.

Over the years, people investigated the concept of *in-situ* resources utilization (ISRU)^4^, so that the expansion and maintenance of lunar bases could rely less on terrestrial materials. ISRU also enables on-site fabrication of massive components of large-scale research and exploration facilities and equipment, such as landing platforms and supports of space telescopes^5^.

A promising locally harvestable resource is lunar regolith. Due to their unique silica-rich structure, lunar soil particles can be thermally fused into solid^6^, yet the part size and power consumption may be the “bottleneck”. Another approach is to use lunar soil as the filler to produce particulate composite. The binder needs to be transported from the Earth. The binder candidates include sulfur^7^, polymer clay composites^8,9^, thermosets^10^, and thermoplastics^11,12^. A major goal of the study in this area is to minimize the binder usage, while the strength and robustness of the material remain satisfactory. In the following description, such a material will be referred to as inorganic-organic hybrid (IOH).

Qiao *et al*.^13^ reported that, due to the irregular particle shapes of lunar soil simulant JSC-1a, even with an optimized particle size graduation, the binder content (*C*) in a regular IOH had to be higher than about 12 wt%; otherwise the filler-binder mixing would be incomplete and the IOH was fragile. A later work indicated that a high compaction pressure could help reduce the binder content to about 8 wt%^14^. Recently, Chen *et al*.^15^ successfully decreased the binder content to only ~4 wt%. The flexural strength of the IOH was 20–40 MPa, much higher than that of typical steel-reinforced concrete. The key technology was the special mixing process. Through extensive mixing and compaction, polymer binder was driven out of the interstitial space among filler particles and was fully utilized to optimize the structural integrity. The ultralow-binder-content lunar-soil-simulant-based IOH is effectively a “lunar cement”. Similar processing procedure may also be applied to other planetary soils or terrestrial sands/soils.

**Figure 1:**
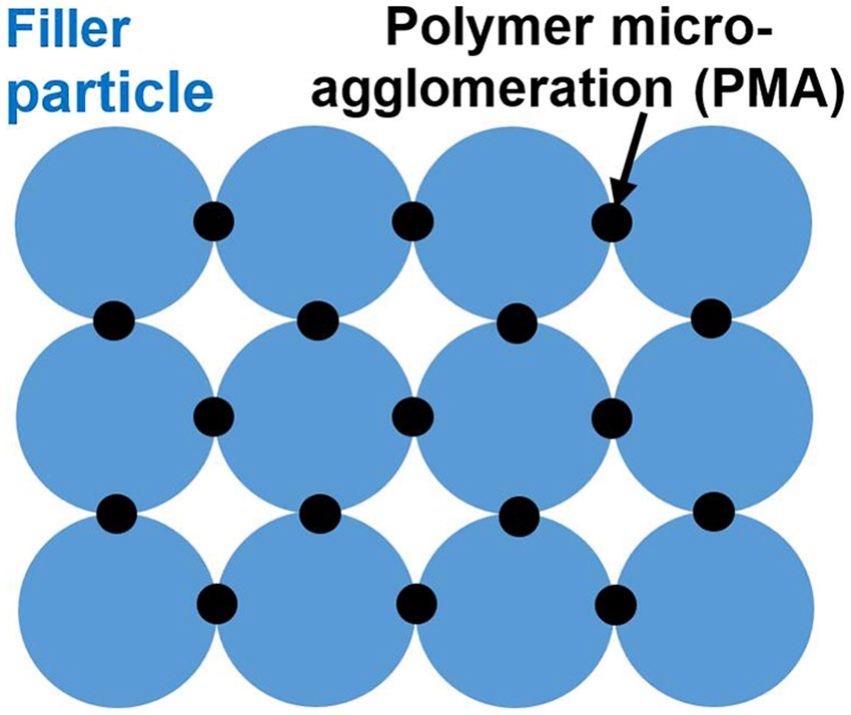
Schematic of ultralow-binder-content IOH “lunar cement”.

The microstructure of IOH “lunar cement” is different from conventional particulate composites. The polymer binder is concentrated at contact places among JSC-1a filler particles, as discontinuous polymer micro-agglomerations (PMA) (Fig. 1). This unique feature would influence many material properties, particularly the fatigue resistance. In a regular composite, the matrix is continuous. Fatigue damage often originates from the surface or the filler-matrix interface, and fatigue cracks propagate in the matrix or along the filler-matrix interface^16^. In the IOH under investigation, on the one hand, PMA is subjected to a relatively large stress concentration, which may promote fatigue damage initiation; on the other hand, fatigue cracks would be trapped by the empty interstitial space among filler particles, which tends to suppress fatigue crack growth. Fatigue resistance measures the long-term reliability of structural materials that work under cyclic loadings. For “lunar cement”, moonquakes may result in prolonged fatigue loadings up to 20 min^17^.

In this paper, two types of samples were designed and tested by different loading setups. The fractography was examined via both optical microscopy and scanning electron microscopy (SEM). Compared with typical steel-reinforced concrete under the same loading condition, IOH can have a better fatigue performance, and may exhibit a typical S-N pattern. Solution environments could compromise fatigue behavior of IOH. The observations present various damage features. A two-step fatigue process was schematized and related to the ultralow binder content.

## Materials and Methods

Processing of IOH “lunar cement” has been discussed in detail in a recent research^15^. All the IOH materials under investigation consisted of 96 wt% filler and 4 wt% epoxy binder. There were two types of IOH: SO, wherein the filler was JSC-1a lunar soil simulant^18^; and SA, wherein the filler was silica sand^19^. The binder was prepared with 5 parts of Epon-828 epoxy resin and 1 part of m-xylylenediamine.

**Figure 2:**
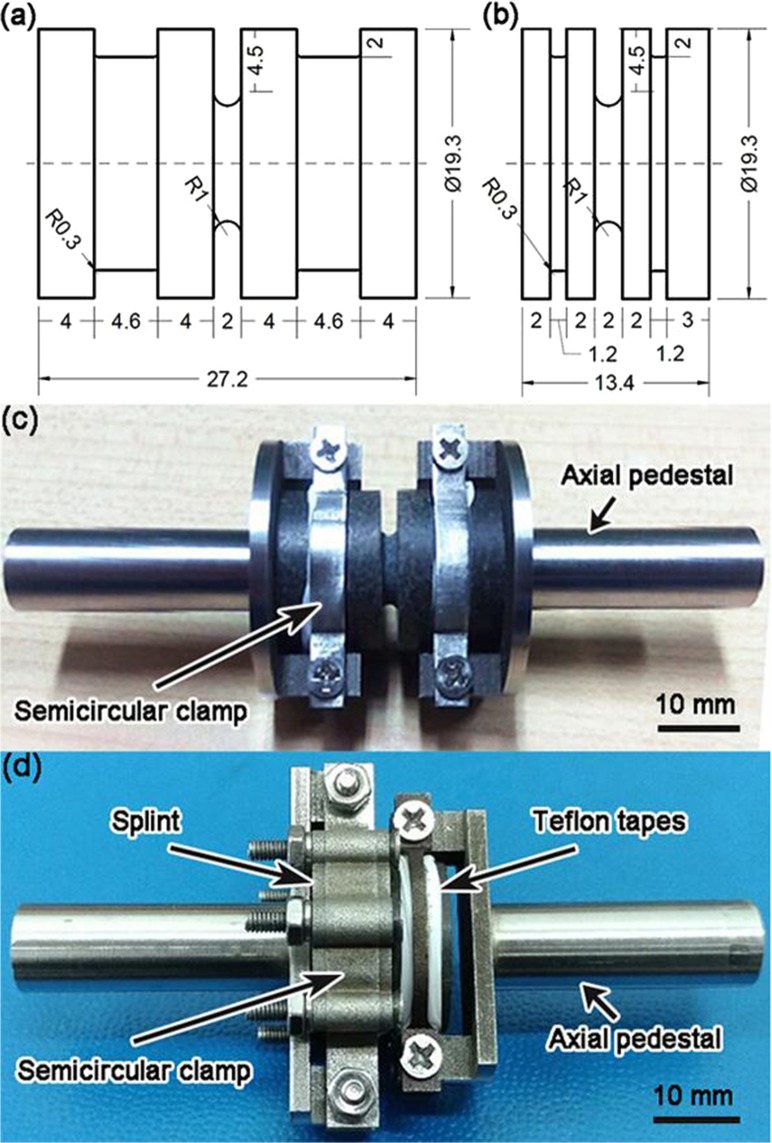
Type I (**a**) and Type II (**b**) fatigue testing samples, all dimensions in mm; and assemblies of Type I (**c**) and Type II (**d**) samples.

Two types of fatigue testing samples, Type I and Type II, were fabricated for rotary-bending experiment. Figure 2(a,b) shows their configurations. Type I sample had a U-shaped notch in the middle and symmetric mounting zones on both ends. Type II sample had a short mounting zone on the loading side and a long mounting zone on the clamping side. Figure 2(c,d) shows the mounting setups. The mounting zones of IOH samples were protected by 150-μm-thick Teflon tapes. The two ends of sample were tightly inserted into two stainless steel holders, respectively. The steel holders had matching holes, secured by semicircular clamps from the exterior. For Type II sample, the holder on the left-hand side (the clamping side) consisted of an axial pedestal, two clamps, and four splints; the holder on the right-hand side (the loading side) was similar to that of Type I sample. All the fixtures were tightened by precision screws.

Fatigue tests were conducted by a Giga-Quad rotary-bending Tester. The magnitude of fatigue loading ranged from 10% to 70% of sample flexural strength. The loading frequency was 16.7 Hz (1000 rpm of the rotation for the tester, which is suitable for the steady operation of the tests) and the stress ratio for this type of loading was −1.

The samples were tested in three different environments: ambient air, fresh water, and 3.5 wt% NaCl aqueous solution. They represent relevant working conditions in lunar bases. In total, five Type I SA samples, fifteen Type II SA samples, and sixteen Type II SO samples were tested. Among them, three Type I SA samples were tested in ambient air; one Type I SA sample was soaked up in water; and one Type I SA was soaked up in NaCl solution. Type II samples were all tested in ambient air. For the water or NaCl solution environment, supplementary liquid was provided through a dripping nozzle during the entire testing procedure, at the rate of about 75 drops per minute. After the fatigue test, fracture surfaces were first coated by a 30-nm-thick copper layer and then examined via optical microscopy and SEM.

## Results and Discussion

**Figure 3:**
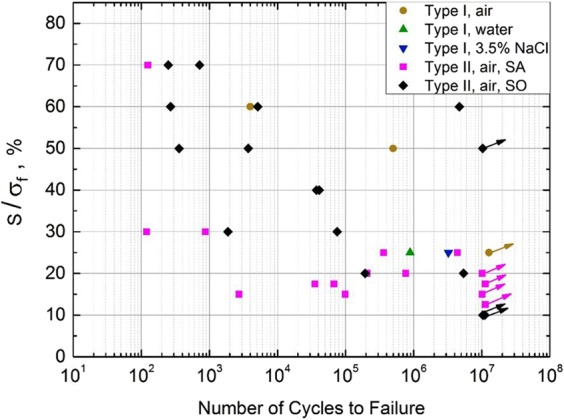
S-N data of IOH samples.

The flexural strengths of SA and SO, *σ*_f_, were measured in three-point bending tests in ambient air, as 39.9 MPa and 47.8 MPa, respectively. The result is consistent with the previous strength measurement of similar materials^15,19^. The measured S-N data are given in Fig. 3.

It can be seen that Type II SA does not clearly exhibit typical S-N curve characteristics. In general, the fatigue life (*N*) of Type II SA ranges from 10^3^ to 10^7^ cycles when *S* is 10–25%*σ*_f_, i.e. ~4 MPa to ~10 MPa; when *S* rises to 70%*σ*_f_ (~28 MPa), the fatigue life is shortened to 10^2^ cycles. In the low stress regime, the correlation between *S* and *N* is quite weak.

Most of the S-N data of Type II SO demonstrate a descending trend: As *S* is 70%*σ*_f_ (i.e. ~33 MPa), *N* is relatively short, around 200 to 10^3^ cycles; when *S* is reduced to 20–40%*σ*_f_ (~10 MPa to ~20 MPa), *N* increases to 10^3^ to 10^7^ cycles; when *S* is 10%*σ*_f_ (~5 MPa), no sample fails at 10^7^ cycles. Two Type II SO samples have excellent fatigue resistance at high stress levels, shown by the two data points at the upper-right corner in Fig. 3: When *S* is 50–60%*σ*_f_ (around 25–30 MPa), *N* ranges from 10^6^ to more than 10^7^.

At the same stress level, the fatigue resistance of SO is better than that of typical portland cement or steel-reinforced concrete^20^, especially when *S* is above 10–15 MPa. The fatigue resistance of SA is somewhat mediocre, comparable with that of typical portland cement at the same stress level. The large data scatter of SA samples, particularly the plateau of S-N data for low stress amplitude, suggests that SA is brittle and does not undergo regular fatigue damage evolution; its failure is dominated by fracture. The relatively well defined S-N trend of SO samples should be associated with fatigue damage accumulation, with “ductile like” features.

The testing data of Type I SA samples suggest that the environmental effect is non-trivial, yet the difference between fresh water and NaCl solution is unclear. When *S* = 25%*σ*_f_ (~10 MPa), the fatigue life of fresh water soaked SA is ~9 × 10^5^ cycles; the fatigue life of NaCl solution soaked SA is ~2 × 10^6^ cycles; the sample tested in air does not fail at 10^7^ cycles. As the liquid soaked epoxy swells^21^, the bonding between epoxy and filler is weakened and therefore, *N* tends to reduce.

The measured fatigue resistance of Type I SA is much better than that of Type II SA. Type I and Type II samples have the same notch depth and cross-sectional size of gauge area. The major difference between them is the mounting zones, which offer different stress concentration factors. The obtained S-N data should be used for self-comparison only. The high stress concentration level of Type II samples suggests that their S-N data are conservative, representing the lower bound of fatigue life. With a more uniform fatigue loading distribution, better fatigue resistance of SO and SA would be measured on Type I samples.

**Figure 4:**
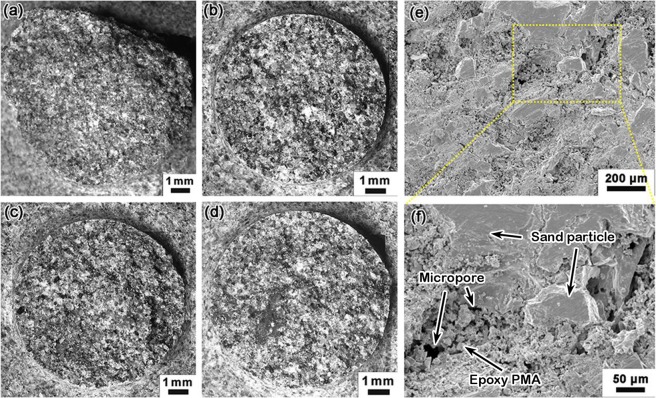
Microscopy of typical failure surfaces of Type II SA samples: (**a**) fracture surface; (**b**) *S* = 30%*σ*_f_, *N* = 8.7 × 10^2^; (**c**) *S* = 15%*σ*_f_, *N* = 2.7 × 10^3^; (**d**) *S* = 25%*σ*_f_, *N* = 4.4 × 10^6^; typical SEM image of Type II SA, prior to fatigue test, in (**e**) low magnification and (**f**) high magnification.

Figure 4(a–d) show the optical microscopy of failure surfaces of Type II SA samples. Figure 4(a) is for a sample fractured in three-point bending. Figure 4(b–d) are for fatigue samples. In all the images, shiny facets of exposed sand filler particles are observed. Since the filler is not adhesive, the IOH strength comes from the binder phase. The filler particles are not damaged during shear or fatigue experiment; the failure is inter-granular. The surfaces demonstrate clear rugged features.

Figure 4(e,f) show the SEM images of Type II SA prior to fatigue test. The fracture surface was generated through three-point bending. The filler particle size ranges from 20 μm to 200 μm, and the discontinuous epoxy PMA are typically 2–5 μm large. The microstructure is porous, since the binder amount is insufficient to fill the interstitial space. Microcracks can be observed occasionally, as a few micropores are connected. The filler particles have been crushed during the high-pressure compaction of IOH processing. The relatively large data scatter in S-N measurement should be attributed to this complex and highly heterogeneous microstructure. Compared with soil particles, sand particles have smoother facets, which tend to weaken the bonding with epoxy; sand particles also have a large number of sharp corners, which act as stress concentrators. Both factors may be responsible for the relatively low fatigue resistance of SA.

**Figure 5:**
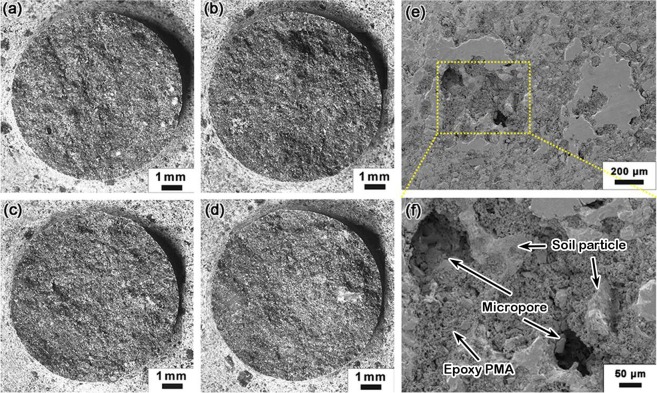
Microscopy of typical failure surfaces of Type II SO samples: (**a**) fracture surface; (**b**) *S* = 70%*σ*_f_, *N* = 2.5 × 10^2^; (**c**) *S* = 40%*σ*_f_, *N* = 4.1 × 10^4^; (**d**) *S* = 20%*σ*_f_, *N* = 5.4 × 10^6^; typical SEM image of Type II SO, prior to fatigue test, in (**e**) low magnification and (**f**) high magnification.

Figure 5 shows the microscopy of failure surfaces of Type II SO samples. Figure 5(a) is for a sample tested in three-point bending; Fig. 5(b–d) are for fatigue samples. No clear rugged patterns are detected, while a few ridges can be observed. Typically, at least one of the ridges spans across the entire cross section. It is interesting that the ridges are randomly oriented, without a common origin, implying that the final failure may be initiated from multiple sites. However, the detailed information of nucleation and propagation of fatigue damage is lost, due to smearing and fragmentation of filler particle clusters. The SEM images as shown in Fig. 5(e,f) also give the microstructural details of Type II SO samples prior to fatigue test. The filler particle size ranges from 20 μm to 200 μm, and the discontinuous epoxy PMA are typically 2–5 μm large, which is similar to those of Type II SA. However, a clear demarcation of the filler particle boundary cannot be perfectly achieved because of the major component – JSC-1a lunar soil simulant, some of which came in the form of powder blending with epoxy PMA. The epoxy-involved soil powders can be properly regarded as a more effective binder than the sparsely scattered epoxy PMA in Type II SA. Thus, Type II SO samples tend to have a relatively better fatigue performance.

**Figure 6:**
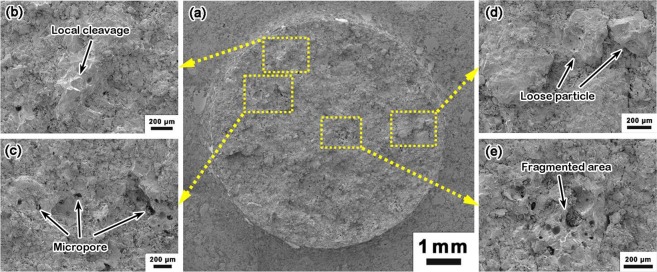
SEM images of a Type II SO sample, fractured upon bending: (**a**) whole cross section; (**b**) cleavage triggered by loose filler particles; (**c**) micropores; (**d**) loose filler particles; and (**e**) fragmented area with micropores.

**Figure 7:**
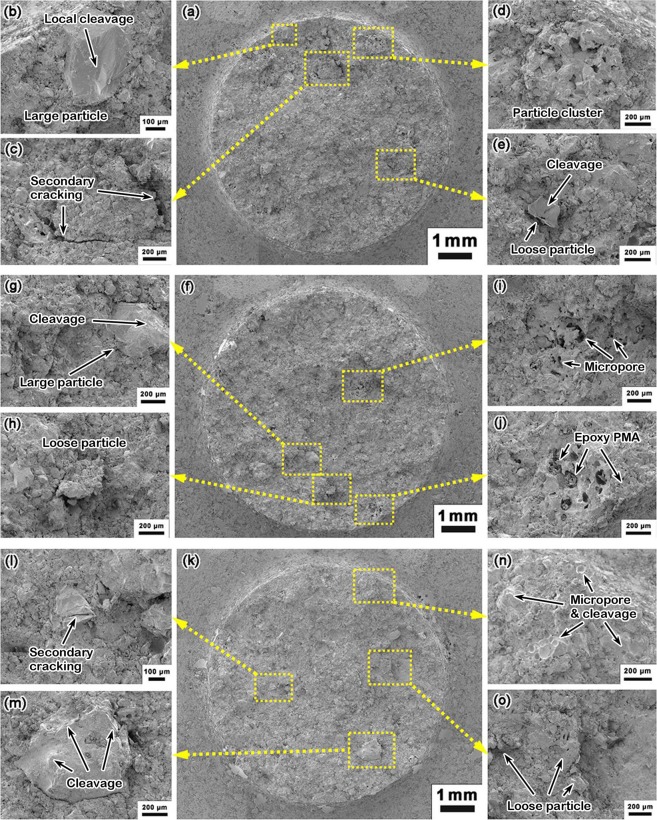
SEM images of a Type II SO fatigue sample (*S* = 70%*σ*_f_, *N* = 2.5 × 10^2^): (**a**) whole cross section; (**b**) cleavage triggered by a large filler particle; (**c**) secondary cracking; (**d**) micropores and a particle cluster; (**e**) cleavage triggered by loose filler particles; (**f**) whole cross section of another Type II SO fatigue sample (*S* = 40%*σ*_f_, *N* = 4.1 × 10^4^); (**g**) cleavage triggered by a large filler particle; (**h**) cleavage triggered by loose filler particles; (**i**) micropores; (**j**) micropores with epoxy PMA; (**k**) whole cross section of another Type II SO fatigue sample (*S* = 20%*σ*_f_, *N* = 5.4 × 10^6^); (**l**) secondary cracking around a large filler particle; (**m**) cleavage triggered in a filler particle; (**n**) micropores and cleavage; (**o**) loose filler particles.

Figures 6 and 7 show the SEM images of Type II SO samples tested under various conditions. Figure 6 is for a sample fractured in three-point bending. Figure 7 is for fatigue samples. It can be seen that the failure process is complicated. While the overall S-N data are “ductile like”, the SEM images show evident local cleavage fracture. The cleavage fracture may be associated with secondary cracking, large filler particles, loose filler particles, filler particle clusters, micropores, and microcracks. Few signs of fatigue crack growth are observed.

It is envisioned that the fatigue damage accumulation in SO occurs mostly in the load-carrying component, the binder phase. Most of the fatigue life is spent on the failure or debond of the binder PMA at critical locations. Because IOH is porous and the PMA size is small, the binder failure is unlikely coupled with any thermal process. Filler particles have important influence on the binder failure. Particularly, at sharp corners, around loose particles, or near large particles, local stress concentration results in faster failure of PMA and eventually, abrupt cleavage-like fracture takes place. The final failure is likely dominated by the connection of distributed early micro-damages in PMA. Collective nucleation and propagation of cracks cause the rugged or ridged markings. These features are distinct from conventional structural materials^22^.

Processing of IOH is still under development. In previous research^15^, we concluded that PMA distribution and filler particle size do not have pronounced influence on the flexural strength of IOH. The S-N data obtained in the current study demonstrate that these two factors are important to the fatigue resistance. If the binder phase is more uniformly distributed or the filler particle size is appropriately gradated, fatigue life of IOH may be much increased.

## Conclusions

Fatigue behavior of inorganic-organic hybrid (IOH) “lunar cement” is investigated through rotary-bending experiment. The IOH is based on lunar soil simulant JSC-1a or sand. The filler particle size is 20–100 microns. The binder content is only 4 wt%. Since the binder is insufficient to fill the gaps among filler particles, the binder phase is discontinuous, with the polymer micro-agglomeration (PMA) size of a few microns.

IOH exhibits unique fatigue damage characteristics. When the filler is sand, the material is brittle and fails when the cycling number is relatively low. When the filler is JSC-1a, the S-N data show “ductile like” trend, while the final failure is dominated by cleavage fracture. The fatigue resistance of JSC-1a based IOH is higher than that of typical steel-reinforced concrete under the same loading condition, especially when the stress amplitude is relatively high. It is likely that most of the fatigue life is spent to debond or break apart binder PMA at critical locations. Once the binder fails, local material is no longer load-carrying and catastrophic failure could be triggered. Such a two-step fatigue process is closely correlated to the ultralow binder content. The binder failure can be promoted by stress concentration around loose filler particles, large filler particles, as well as sharp corners of filler particles. When the sample is soaked up by water or sodium chloride solution, the fatigue resistance significantly decreases, which should be attributed to the swelling of PMA.

The current study offers important guidance to the further development of IOH. While the flexural strength is quite insensitive to the binder distribution and the filler particle size, these two factors may be critical to fatigue resistance. This finding has important relevance to *in-situ* resource utilization for lunar exploration missions.
